# The Role of Endocan in Selected Kidney Diseases

**DOI:** 10.3390/ijms21176119

**Published:** 2020-08-25

**Authors:** Magdalena Nalewajska, Klaudia Gurazda, Małgorzata Marchelek-Myśliwiec, Andrzej Pawlik, Violetta Dziedziejko

**Affiliations:** 1 Department of Nephrology, Transplantology and Internal Medicine, Pomeranian Medical University, Powstańców Wlkp 72 Str, 70-111 Szczecin, Poland; magda_nalewajska@yahoo.co.uk (M.N.); malgorzata.marchelek@gmail.com (M.M.-M.); 2 Department of Biochemistry and Medical Chemistry, Pomeranian Medical University, Powstańców Wlkp 72 Str, 70-111 Szczecin, Poland; gurazdaklaudia@gmail.com (K.G.); viola@pum.edu.pl (V.D.); 3 Department of Physiology, Pomeranian Medical University, Powstańców Wlkp 72 Str, 70-111 Szczecin, Poland

**Keywords:** endocan, ESM-1, acute kidney injury, chronic kidney disease, renal replacement therapy, kidney transplantation

## Abstract

Endocan, previously referred to as an endothelial-cell-specific molecule-1 (ESM-1) is a member of a proteoglycan family that is secreted by vascular endothelial cells of different organs, mainly lungs and kidneys. It is assumed to participate in endothelial activation and the triggering of inflammatory reactions, especially in microvasculatures. Thanks to its solubility in human fluids, i.e., urine and blood plasma, its stability and its low concentrations in physiological conditions, endocan has been proposed as an easily available, non-invasive biomarker for identifying and predicting the course of many diseases. Recently, endocan has been studied in relation to kidney diseases. In general, endocan levels have been linked to worse clinical outcomes of renal dysfunction; however, results are conflicting and require further evaluation. In this review, authors summarize available knowledge regarding the role of endocan in pathogenesis and progression of selected kidney diseases.

## 1. Introduction

Endocan, formerly known as endothelial-cell-specific molecule-1 (ESM-1) is a soluble dermatan sulphate proteoglycan with a molecular mass of 50 kDa that is expressed and secreted into the bloodstream from vascular endothelial cells, mainly of lungs and kidneys [1,2]. Its expression is upregulated either by proangiogenic factors, such as vascular endothelial growth factor (VEGF), fibroblast growth factor 2 (FGF-2) or proinflammatory cytokines, like tumor necrosis factor alfa (TNFalfa), interleukin-1beta, hypoxia-inducible factor-1alfa, and lipopolysaccharide, whereas it is downregulated by interferon gamma [2]. It has been reported that endocan is involved in the rearrangement of endothelial cells, namely cell adhesion, migration, proliferation and angiogenesis, as well as playing a crucial role in inflammatory processes [3]. Nonetheless, studies provide conflicting results regarding the biological role of endocan in pathological processes. Some authors report that endocan upregulates cell adhesion molecules—ICAM-1 (intracellular cell adhesion molecule-1), VCAM-1 (vascular cellular adhesion molecule-1) and E-selectin—or activates the nuclear factor-kappa B pathway, an important mediator in inflammatory processes; therefore, endocan could take part in vascular inflammation and endothelial cell activation [4]. Contrarily, endocan could display anti-inflammatory properties by blocking the recruitment, adhesion and activation of leukocytes through direct binding to LFA-1 (leukocyte function-associated antigen 1) and interference with the LFA-1/ICAM-1 pathway [5].

Despite these conflicting results, the characteristics of endocan—its extremely low concentrations in physiological conditions, its stability, and its easy and non-invasive detection in body fluids—suggest endocan as a possible biomarker of clinical importance [6,7]. Endocan has already been linked to the occurrence and course of various conditions, including inflammatory diseases, cancer, sepsis, cardiovascular events (CVE) and kidney diseases [1,7,8,9].

So far, endocan has been studied in acute (AKI) and chronic kidney disease (CKD) and renal replacement therapy (RRT). In this paper, authors review available research concerning the role of endocan in the development and progression of selected kidney diseases. The most valuable studies concerning the role of endocan in kidney diseases have been summarized in Table A1.

## 2. Endocan in AKI

AKI is a theoretically reversible condition characterized by a rapid reduction in renal function that could lead to the accumulation of nitrogen-based end-products or a decline in urine output [10]. AKI is diagnosed upon one of the following definitions: an increase in serum creatinine levels by either ≥0.3 mg/dL within 48 h or ≥1.5-fold from a known or assumed baseline, or a reduction in urinary output by less than 0.5 mL/kg/h over 6 h [10]. Since inflammation and endothelial dysfunction play a part in the pathogenesis of AKI, it has been assumed that endocan could reflect renal dysfunction in this group of patients. However, little data is available covering the role of endocan in AKI.

In 2015, Rahmania et al. assessed the predictive value of endocan in relation to acute renal failure and the need for RRT in a group of intensive care unit (ICU) patients admitted due to the diagnosis of acute respiratory distress syndrome (ARDS) [11]. In total, 17% of the 96 subjects required RRT at some point during hospitalization, and these patients displayed higher plasma endocan and creatinine levels than those who did not need RRT. ROC AUC identified combined serum endocan and creatinine levels as the most valuable mean for predicting the need for RRT, in comparison to either creatinine or endocan alone. Gunay et al. published the results of a study evaluating the levels of endocan in the serum of patients diagnosed with AKI [12]. Among other parameters, serum endocan, creatinine and blood urea nitrogen were higher in the AKI group than in healthy subjects. The ROC analysis further displayed the high sensitivity and specificity (59% and 76.3%, respectively) of endocan plasma measurements in the identification of AKI. The authors, however, did not clarify the renal clearance of endocan and, thus, were not able to evaluate whether endocan levels in AKI were increased following its enhanced production or decreased elimination. p14, endocan peptide cleavage of 14 kDa was recently identified as a product of endocan proteolysis by cathepsin G and was evaluated in sepsis [13]. Gaudet et al. conducted a post hoc analysis of the data from previous research of septic patients in relation to p14 and renal function [14,15]. Authors calculated the plasma endocan cleavage ratio (ECR) using the following equation: Plasma p14/(endocan + p14), and the results were expressed in pmol/mL. The ECRs were measured at baseline and 24, 48, and 72 h after admission to the ICU. The results demonstrated that the renal component of the SOFA scale (sequential organ assessment score) was related to an increased ECR at baseline. At 72 h, patients with a SOFA scale >4 at enrolment displayed significantly higher ECR than those with a score <4. The authors suggest that plasmatic levels of p14 could rely upon renal function. Measurements of p14 in urine could be of more value as p14 is supposed to be eliminated through glomerular filtration due to its smaller molecular weight and the lack of a polyanionic glycanic chain.

It has been also hypothesized that examining serum endocan levels could help differentiating between various pathogenesis of intrinsic AKI [6]. Following the considerations of Azimi [6], intrinsic causes of AKI could be divided into two large groups: Tubular and glomeruli/vasculatures injuries. The first group is represented by ischemic/toxic tubular injuries and tubulointerstitial pathologies, whereas the other one by i.e., glomerulonephritis and vasculitis. Endocan as a marker of endothelial disfunction, a disfunction that is an important contributor to glomeruli/vasculatures pathologies, and less to tubular and tubulointerstitial diseases of the kidney could serve as a useful tool in diagnostics of these two entities [6].

## 3. Endocan in CKD

CKD is defined as a decreased glomerular filtration rate (GFR) of less than 60 mL/min/1.73 m^2^ or damaged kidney structure, identified by imagining studies or renal biopsy, which occurs for more than 3 months. Diabetes mellitus, primary glomerulonephritis and hypertension (HT) are listed as the three main causes of CKD. The course of CKD is associated with a gradual loss of kidney function that can eventually result in the need of RRT or renal transplantation, and end-stage renal disease (ESRD) itself is linked to increased mortality, particularly from cardiovascular diseases [16]. As mentioned previously, endocan has been proven to display a prognostic value in CKD, among other conditions [8], as reduced kidney function is linked to endothelial dysfunction and inflammation [17].

In a study by Yilmaz et al., the serum concentrations of endocan were assessed in stage 1–5 CKD patients in the pre-dialysis period in relation to inflammation, endothelial dysfunction, cardiovascular incidence and overall survival [9]. The results showed that patients with CKD displayed higher plasma endocan levels than controls, and the concentrations of endocan correlated positively with CKD stage and negatively with estimated glomerular filtration rate (eGFR). Plasma endocan levels further correlated positively with inflammatory markers, such as hsCRP (high-sensitivity C-reactive protein) and PTX3 (pentraxin 3), and carotid-intima media thickness. Cox survival analysis also demonstrated the positive association between endocan plasma concentrations and all-cause mortality and CVE in CKD patients. Further analysis also displayed an increased predictive value of endocan in relation to CVE in CKD subjects compared with the usual risk factors. Following the results of other studies, the authors explained that endocan could affect vascular inflammation by interacting with ICAM-1LFA-1 and leukocyte extravasation [5]. Pawlak et al. evaluated the role of endocan in CKD patients with CVE [18]. In their study, serum endocan levels, serum levels of soluble ICAM-1 and VCAM-1 and inflammatory markers were significantly elevated in the CKD group compared to controls. In contrast to a study by Yilmaz et al. [9], the authors did not display correlation between endocan and markers of kidney function, suggesting that decreased clearance does not influence the endocan levels [18]. Furthermore, endocan, sICAM-1, sVCAM-1 and the majority of inflammatory marker concentrations were higher in CKD in the CVE group than in the group of CKD without CVE [18]. Samouilidou et al. recently studied the association between serum endocan levels and lipid profiles of CKD patients with dyslipidemia, dividing patients into non-dialyzed and HD subgroups [19]. Endocan concentrations were also verified in relation to two members of the paraoxonase family, PON1 and PON3, which contribute to HDL-related antiatherogenic properties due to the inhibition of LDL oxidation. The results of the study indicated that endocan serum levels were significantly higher in the HD subgroup compared to others; PON1 levels were decreased in the HD group, whereas PON3 levels were increased in both studied subgroups compared to controls. The endocan levels correlated positively with total cholesterol and LDL-C in both studied groups and inversely with HDL-C in hemodialyzed patients. Furthermore, endocan correlated with PON1 in both CKD groups. The authors suggest that the elevated endocan levels in HD patients may be related to increased endocan production due to the atherosclerotic state, as indicated by the lowered HDL-C levels in this group. Moreover, the decrease in PON1 levels in HD subjects might be the main factor influencing the elevated endocan levels in the mentioned group.

Another research group investigated the role of endocan and another glycoprotein, endoglin, in CKD caused by diabetes mellitus [20]. The study included diabetic patients with and without diabetic nephropathy (DN), and controls. The results indicated that both endocan and endoglin levels were higher in diabetes mellitus patients than in the control group. Moreover, patients with microalbuminuria displayed increased endocan plasma concentrations in comparison to normoalbuminuric diabetic patients and controls, whereas endoglin concentrations in DN patients were higher only compared to healthy subjects. Therefore, the authors concluded that endocan could serve as a potential marker of microvascular complications of diabetes mellitus. In the study conducted by Arman et al., diabetic patients with no other inflammatory diseases displayed higher endocan serum concentrations than the control group [21]. After 3 months of lifestyle modifications and pharmacological treatment of diabetes, the endocan levels and albuminuria decreased in the studied group. Additionally, results showed a positive correlation between the decrease in endocan and albuminuria levels. On the other hand, in a study performed by Cikrikcioglu et al., patients with macroalbuminuria in the course of diabetes mellitus surprisingly showed lower serum endocan levels in comparison to normoalbuminuric and microalbuminuric patients [22]. Moreover, the urine albumin-creatinine ratio (UACR) and urine protein-creatinine ratio (UPCR) displayed negative correlation with endocan levels. No correlation was established between serum endocan levels and duration of diabetes, serum creatinine and eGFR. The authors hypothesized that the obtained results could occur due to the VEGF-related mechanism. In the hyperglycemia state, VEGF is responsible for abnormal angiogenesis and enhanced permeability of blood vessels and is also supposed to stimulate endocan release [23]. Since podocytes and renal tubular cells are responsible for VEGF secretion [23], the increasing renal injury with the clinical manifestation of macroalbuminuria leads to decreased VEGF expression and, therefore, to decreased plasma endocan levels [24,25]. Zheng et al. studied the glomerular transcriptomes from murine strains of different DN susceptibility in order to identify genes that are responsible for DN occurrence [26]. Authors recognized the ESM-1 gene as a DN resistance factor. In the study, the glomerular ESM-1 expression correlated negatively with DN susceptibility and also inhibited leukocyte infiltration, one of the pathophysiological processes that occurs in DN. Moreover, urine ESM-1 significantly increased with diabetes in the DN-resistant strain in vivo, indicating that urine ESM-1 could serve as a non-invasive biomarker of glomerular ESM-1.

Oktar et al. have recently evaluated plasma endocan levels in patients with newly diagnosed HT [27]. Significantly elevated endocan levels in HT have already been reported in previous studies [28,29]. However, the study by Oktar et al. was the first to indicate that microalbuminuria is elevated in patients with newly diagnosed HT and that it positively correlates with endocan concentrations.

In another study, the relevance of endocan levels in IgA nephropathy (IgAN) has been verified [30]. Both plasma and urine concentration have been significantly increased in the IgAN group compared with controls. The levels of plasma endocan did not differ among CKD stage groups; however, the urine endocan levels increased in the advanced CKD groups. Also, both plasma and urine endocan levels were increased in the advanced pathologic grades in kidney biopsies according to the Lee classification, but not the Oxford classification. Furthermore, high plasma endocan concentrations were assessed as an independent risk factor for renal function decline. Authors suggest that elevated plasma endocan levels could be secondary to pathologic changes and endothelial damage, whereas increased urine concentrations may occur due to a damaged glomerular basement membrane.

The role of endocan, among other molecules associated with endothelial function, has also been evaluated in a study of ADPKD (autosomal dominant polycystic kidney disease) patients [31]. The subjects included in the study were divided into the following groups: ADPKD patients with impaired renal function, ADPKD patients with preserved renal function and matched control group. Patients included in the first group had significantly increased levels of endocan in comparison to the second group and the controls. Concomitantly, ADPKD patients with normal renal function displayed higher endocan levels than the control group. The results also showed an inverse correlation of endocan and eGFR. In this study, the authors indicated that ADPKD pathogenesis could also depend upon impaired endothelial function, increased angiogenesis and hypoxia.

## 4. Endocan in RRT

ESRD requires RRT—HD, peritoneal dialysis (PD) or kidney transplantation (KT). KT is the method of choice in the treatment of ESRD; however, progressive graft function still remains a clinical challenge. Identification of new biomarkers that would enable timely diagnosis and management of graft function deterioration is of great importance.

In 2012, Li et al. studied the significance of dynamic monitoring of endocan expression in the serum and renal allograft tissues of kidney transplant recipients diagnosed with an acute rejection (AR) [32]. Despite improved short-term graft function due to advanced immunosuppressants, ARs can still contribute to chronic allograft dysfunction. In the presented study, endocan expression was elevated in the blood and allograft tissues in ARs compared to patients with normal allograft function and allograft dysfunction from other causes. The anti-rejection treatment was able to decrease its expression. As expected, endocan mRNA in peripheral blood was increased in patients with acute vascular rejection in comparison to acute cellular rejection. Authors point out the damaged endothelium of the donor kidney as the source of endocan in AR patients. They further inform that endocan could serve as a sensitive and specific marker for AR detection, and its value could even increase when combined with assessment of urine HLA-DR+ lymphocytes. Lee et al. further evaluated the role of endocan as a diagnostic marker for antibody-mediated rejection (AMBR) in patients after KT [33]. The AMBR manifests as vascular inflammation—glomerulitis and peritubular capillaritis—and is related to endothelial dysfunction. In the mentioned study, endocan levels in the serum and urine were higher in patients with AMBR than with other kidney allograft pathologies (acute tubular necrosis, BK virus associated nephropathy, T-cell mediated rejection). Endocan levels correlated positively with scores of glomerulitis and peritubular capillaritis but neither with tubulitis nor interstitial inflammation severity, indicating the endothelial origin of endocan. Moreover, patients with AMBR and elevated urine and plasma endocan levels displayed worse renal function despite its function at baseline. The main finding of this research is that evaluation of both urine and serum endocan levels could serve as a diagnostic tool for vascular inflammation in kidney transplant recipients and could be convenient in distinguishing between AMBR and other allograft pathologies. Another cross-sectional study has been conducted to assess the relationship between serum endocan levels and severity of chronic graft dysfunction in kidney transplant recipients [8]. Serum endocan levels correlated with CKD stage, and patients with higher endocan levels displayed higher creatinine and lower GFR levels than patients with lower endocan concentrations in the 3-month follow-up. Furthermore, endocan correlated with TNFα, a cytokine participating in endothelial activation. In vitro stimulation of endothelial cells with TNFα resulted in increased production of endocan and TGF-β1, and reduced IL-10 expression. The authors concluded that TNFalfa shows bidirectional properties in maintaining immune balance in kidney transplant recipients—it contributes to elevated leucocyte recruitment at inflammatory sites in endocan-mediated pathways but displays anti-inflammatory properties through TGF-β1 activity. In a study by Malyszko et al., endocan plasma concentrations were significantly elevated in kidney transplant recipients with stable graft function compared to healthy subjects [34]. Endocan levels correlated positively with other markers of endothelial dysfunction (i.e., ICAM and VCAM), with creatinine levels, and inversely with eGFR. Further analysis indicated creatinine, ICAM and VCAM as predictors of endocan expression. Authors conclude that endocan levels reflect the degree of endothelial damage and, therefore, it may play a role as a marker of graft injury and graft function deterioration. De Souza et al. studied serum endocan concentrations in pediatric patients 6–24 months after KT, in relation to HT and renal graft function [35]. Patients with HT and CKD displayed higher endocan plasma levels than those without HT and CKD, and these levels correlated positively with systolic blood pressure and pulse pressure and negatively with eGFR. The cut-off point of 7.0 ng/mL of endocan concentrations managed to identify children with HT and CKD with 100% sensitivity and 75% specificity.

Endocan has also been studied recently in PD patients. Oka et al. assessed correlation between serum endocan levels and the extent of urine decline in 21 PD patients [36]. Decline in urine volume is one of the main negative consequences of PD, which leads to disrupted fluid control. It mainly results from decreased residual renal function. In the study, the serum endocan levels were increased in PD patients compared to controls. They also positively correlated with proteinuria, serum creatinine, TNFalfa, beta2-macroglobulin level and PD drainage volume; there was no correlation between endocan and urine volume at baseline. However, further analysis identified serum endocan and proteinuria levels at baseline as predictors of the extent of urine decline in the studied group. Poon et al. [37] studied the relationship between endocan levels in the serum of PD patients and clinical outcomes. The authors hypothesized that endothelial injury may influence the vascular physiology and peritoneal transport in PD; therefore, endocan may be useful as a prognostic marker in this group of patients. The results of the study indicated the negative correlation between serum albumin and endocan levels, the progressive decrease in subjective global assessment scale and an increase in comprehensive malnutrition-inflammation score in relation to endocan; thus, it may play a role in the nutritional status of PD patients. The endocan levels also positively correlated with CRP levels and arterial stiffness markers, which is consistent with previous studies. Poon et al. were, however, the first to reveal that higher serum endocan levels are linked to worse cardiovascular event-free survival in PD patients with uncontrolled blood pressure.

## 5. Conclusions

Non-invasive diagnosis of various kidney diseases remains a challenge in clinical practice. Serum creatinine level, blood urea nitrogen and proteinuria are among the commonly practiced diagnostic means to evaluate kidney pathologies; however, they are not specific or immediate, and are imprecise in predicting renal function. There is emerging evidence linking endocan and the identification and outcomes of AKI, CKD and kidney transplantation, thereby bringing hope for a novel, non-invasive diagnostic marker. Nevertheless, the results of the conducted studies are conflicting. Since kidney diseases display high diversity in terms of pathogenesis and clinical course, the exact mechanisms by which endocan could affect kidney functions is yet to be determined. The issue as to whether serum endocan levels are a consequence of increased production or decreased renal clearance, and also whether the origin of urinary endocan lies upon its secretion by damaged renal tubular cells or its leakage from plasma through disrupted glomerular basement membrane is still to be clarified. The hypothesis that proposes endocan as an anti-inflammatory mediator in terms of disruption of lymphocytes adhesion to endothelium, thereby mediating immune balance in kidney diseases, is also of great interest, however, still needs further evaluation. Further analyses are mandatory before endocan could be used as a diagnostic tool in clinical practice.

## Appendix A

**Table A1:** Most valuable studies examining the role of endocan in various kidney diseases.

Condition	Aims	Study Group	Results	References
AKI	Correlation between endocan levels and renal function and need for RRT in ARDS patients in ICU	96 patients with ARDS in ICU department who did not require RRT at baseline	Serum creatinine and endocan together—most valuable in predicting the need for RRT (ROC AUC 0.77)	[11]
AKI	serum endocan levels as potential biomarkers for diagnosing AKI and its pathogenesis	39 patients diagnosed with AKI according to KDIGO definition	Endocan displayed 59% of sensitivity and 76.3% of specificity in the diagnosis of AKI	[12]
CKD	Endocan levels in CKD and non-CKD patients; relationship between endocan levels and inflammatory and endothelial dysfunction markers in CKD; endocan as predictive marker of all-cause mortality and CVE in CKD	251 CKD pre-dialysis patients (23.1% DM; 18.7% HT; 15.9% chronic glomerulonephritis; 27.1% unknow etiology)	CKD patients displayed higher plasma endocan levels than controls; plasma endocan correlated with eGFR, different markers of inflammation and vascular abnormalities (FMV and CIMT); endocan levels were associated with all-cause mortality and CVE independent of traditional risk factors	[9]
DN	Predictive role of endocan and endoglin as markers of DN progression	96 patients with DM2 (40 patients with normoalbuminuria, 56 patients with DN)	Endocan and endoglin serum levels were higher in DM2 patients; endocan, but not endoglin levels were higher in DN compared to normoalbuminuric patients (*p* = 0.011 and *p* = 0.822, respectively)	[20]
IgAN	Relevance of plasma and urine endocan levels in IgAN	64 patients with IgAN diagnosed upon renal biopsy	Urine and plasma endocan levels were significantly higher in IgAN group than controls; urine, but not plasma endocan correlated with CKD stage; high plasma endocan was an independent risk factor for CKD progression	[30]
AR after KT	Dynamic monitoring of serum endocan levels in diagnosing ARs	60 patients after KT (20 with normal renal function, 20 with biopsy-proven AR, 20 with renal allograft dysfunction from other causes)	Elevated blood and tissue expression of endocan in the AR group compared to others	[32]
AR after KT	Endocan as marker of microvascular inflammation in KT patients	203 patients after KT	Increased urine and plasma endocan levels in AMBR patients than others; higher scores of microvascular inflammation in biopsy specimens and worse renal survival in patients with increased endocan levels (urine and/or plasma); serum and urinary endocan were valuable in distinguishing between AMBR and other graft pathologies	[33]

AKI—acute kidney injury; RRT—renal replacement therapy; ARDS—acute respiratory distress syndrome; ICU—intensive care unit; CKD—chronic kidney disease; CVE—cardiovascular events; DM—diabetes mellitus; HT—hypertension; eGFR -estimated glomerular filtration rate; FMV—flow-mediated vasodilation; CIMT—carotid intima media thickness; DN—diabetic nephropathy; IgAN—IgA nephropathy; AR—acute rejection; KT—kidney transplantation; AMBR—antibody-mediated acute rejection.
